# Use of hare bone for the manufacture of a Clovis bead

**DOI:** 10.1038/s41598-024-53390-9

**Published:** 2024-02-05

**Authors:** Todd A. Surovell, McKenna L. Litynski, Sarah A. Allaun, Michael Buckley, Todd A. Schoborg, Jack A. Govaerts, Matthew J. O’Brien, Spencer R. Pelton, Paul H. Sanders, Madeline E. Mackie, Robert L. Kelly

**Affiliations:** 1 Department of Anthropology, University of Wyoming, Laramie, WY USA; 2 Office of Archaeology and Historic Preservation, History Colorado, Denver, CO USA; 3 Manchester Institute of Biotechnology, The University of Manchester, Manchester, UK; 4 Department of Molecular Biology, University of Wyoming, Laramie, WY USA; 5 Department of Anthropology, Chico State University, Chico, CA USA; 6 Office of the Wyoming State Archaeologist, Department of Anthropology, University of Wyoming, Laramie, WY USA; 7 Department of Sociology and Anthropology, Weber State University, Ogden, UT USA

**Keywords:** Archaeology, Proteomics

## Abstract

A tubular bone bead dating to ~ 12,940 BP was recovered from a hearth-centered activity area at the La Prele Mammoth site in Converse County, Wyoming, USA. This is the oldest known bead from the Western Hemisphere. To determine the taxonomic origin of the bead, we extracted collagen for zooarchaeology by mass spectrometry (ZooMS). We also used micro-CT scanning for morphological analysis to determine likely skeletal elements used for its production. We conclude that the bead was made from a metapodial or proximal phalanx of a hare (*Lepus sp.*). This find represents the first secure evidence for the use of hares during the Clovis period. While the use of hare bone for the manufacture of beads was a common practice in western North America during the Holocene, its origins can now be traced back to at least the terminal Pleistocene.

## Introduction

The production and use of personal ornaments, most commonly beads, are important indicators of increasing human cultural and social complexity in the Paleolithic, appearing first in the Middle Stone Age of Africa and later in the Early Upper Paleolithic of Eurasia^1–6^. Although beads are not as well documented from early archaeological contexts in the Americas, several examples have been reported from Paleoindian localities indicating that the first migrants to the Western Hemisphere made and used personal ornamentation, whether to decorate their bodies and/or clothing^7–15^.

Relatively few beads have been recovered from secure Early Paleoindian contexts. For example, a poorly dated caliche bead was recovered from Pleistocene sediments in a core at the Mockingbird Gap site in New Mexico^7^, and four hematite beads were found by an avocational archaeologist within what is likely a Clovis age burial in Colorado^9^. Bone beads are known but from slightly later Younger Dryas-aged contexts at the Lindenmeier^13^, Powars II^8^, and Hell Gap^14^ sites. In this paper, we report the material used for the manufacture of a ~ 12,940 year old tubular bone bead from the La Prele Mammoth site in Converse County, Wyoming, USA. We suggest that this bead is among the oldest, if not the oldest, known ornament from the Americas with one possible exception^16^.

For taxonomic identification of the animal from which bone was derived, we turned to zooarchaeology by mass spectrometry (ZooMS). ZooMS takes advantage of differences in the primary structure of the collagen protein to identify the familiar, generic, or specific origin of archaeological bone fragments^17,18^. After collagen is digested using the enzyme trypsin, the masses of the resulting peptides are measured using matrix assisted laser desorption/ionization time of flight mass spectrometry (MALDI-TOF MS). This process generates a protein mass fingerprint that can be compared to fingerprints of known taxa. When collagen is well preserved, the ZooMS method can be used to identify highly fragmented faunal assemblages for which traditional morphological identification is challenging^19–22^. ZooMS can also identify taxa used to produce bone tools^23–27^. For this reason, it is an ideal method for identifying the taxonomic origins of the bead from La Prele.

**Figure 1:**
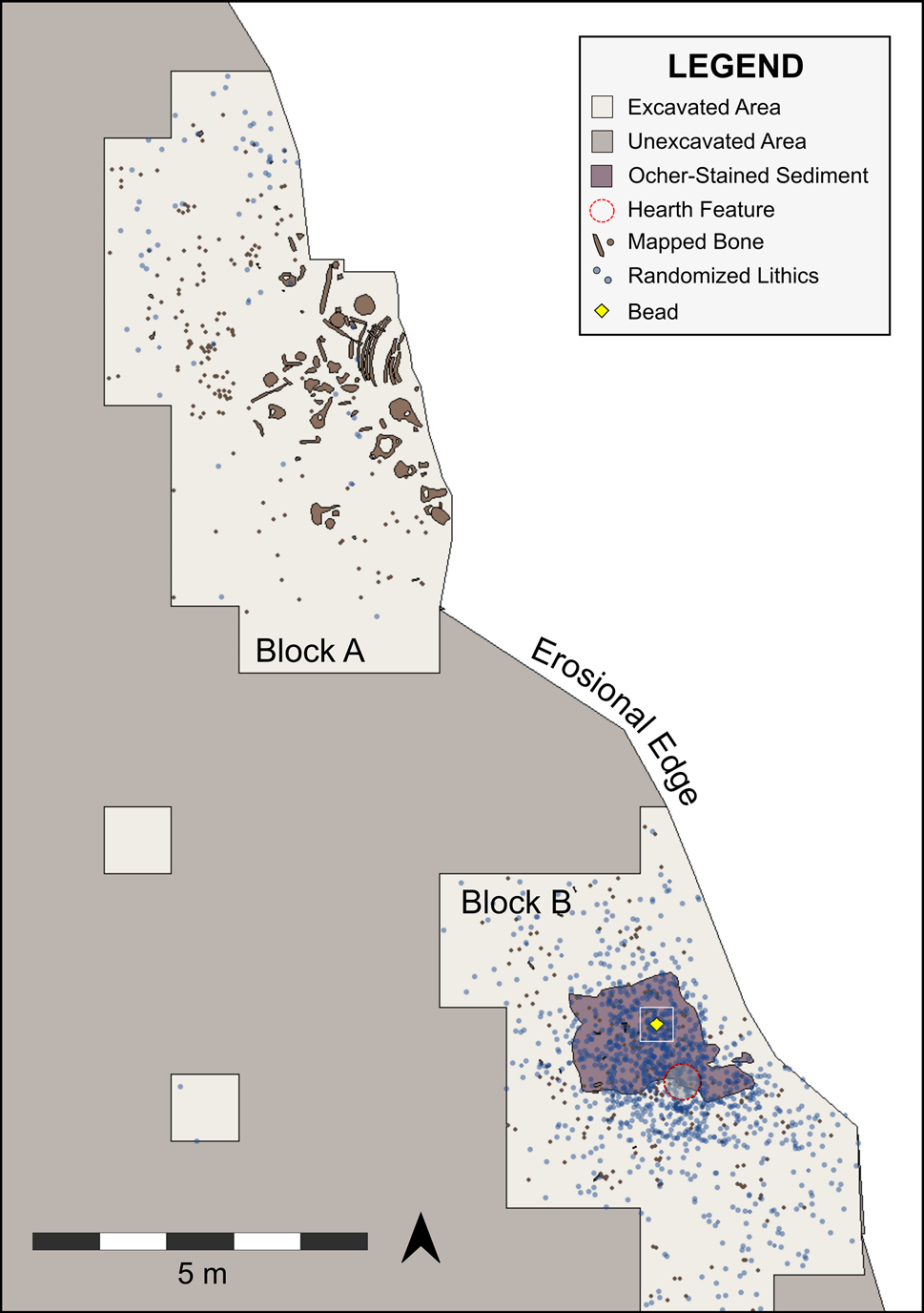
Plan map of a portion of the La Prele Mammoth site showing the location of the bone bead (yellow diamond) in excavation Block B. Chipped stone shown as total counts randomized within 50 × 50 cm excavation quads.

The La Prele Mammoth site is an Early Paleoindian site in Converse County, Wyoming along La Prele Creek near its confluence with the North Platte River (Supplementary Fig. 1)^28,29^. Test excavations by Frison in 1987 revealed the association of chipped stone artifacts with the partial remains of a subadult Columbian mammoth (*Mammuthus columbi*), and later excavations identified a nearby camp area preserving multiple hearth-centered activity areas. The occupation surface was buried by low energy overbank deposits, and based on the average of five radiocarbon dates on bone, the occupation occurred at 12,941 ± 56 cal yr BP^30^. The bead was recovered from Block B, a hearth-centered activity area approximately 11 m south southeast and upstream of the mammoth. This part of the site contained a zone of hematite- or red ochre-stained sediment that was truncated by erosion on its eastern edge. The remaining portion of the red ochre stain spanned 3.2 m^2^ with a hearth on its southern edge. The ochre has been geochemically sourced to the vicinity of the Powars II site, a well-documented location of Paleoindian hematite quarrying, 85 km to the southeast^8,31–33^. From Block B, over 1,000 pieces of chipped stone have been recovered including seven flake tools. Several fragments of eyed bone needles were also recovered. This area produced a faunal assemblage consisting mostly of butchered and burned remains of *Bison antiquus*. Distance-decay and ring and sector analyses suggest that the hearth sat within a structure approximately 3.3 m in diameter^34^. We recovered the bead from screened (1/16 in. mesh) sediments within the ochre stain from a 50 × 50 cm excavation quadrant approximately 1 m northwest of the hearth’s center (Fig. 1).

**Figure 2:**
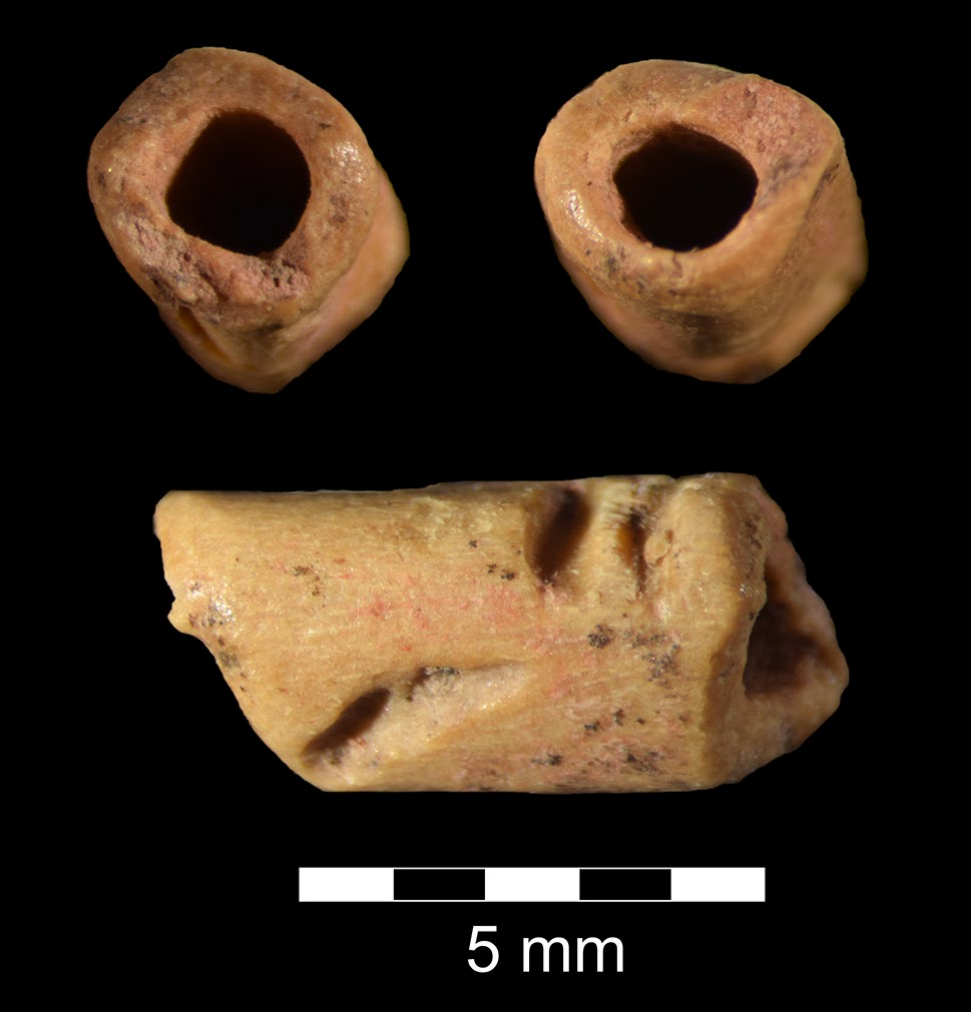
La Prele bone bead showing polished ends (upper) and side view with incisions (lower).

The bead is small, approximately 7 mm in length. Its internal diameter averages 1.6 mm, and it has a mean external diameter of 2.9 mm. Two deep parallel grooves with U-shaped cross-sections occur on the face of the bead aligned perpendicular to its long axis (Fig. 2; Supplementary Fig. 2; Supplementary Video 1). An oblique groove of similar size and morphology occurs closer to the other end of the bead and on a different rotational face. Whether these incisions are byproducts of manufacture, skinning, wear, or possibly decorations is not known, but similar grooves occur on Paleolithic and Archaic tubular bone beads^35,36^. Both ends of the bead are highly smoothed and polished. Although the bead is lightly coated in red ochre, the presence of ochre on its surface might be incidental as it was recovered from sediments that were stained by powdered hematite.

We considered the possibility that the bead is not of human manufacture but instead the product of carnivore consumption and digestion, as bone tubes of small mammals are sometimes found in carnivore scat, and they can show characteristics exhibited by the bead^37,38^. Digestive pitting on long bone shaft fragments and polishing on fracture surfaces can occur on bone fragments passed by coyotes (*Canis latrans*)^37,38^ and grooves on the bead surface are similar in size and shape to ‘scores’ produced by carnivore gnawing^39^. We find the carnivore hypothesis unlikely for five reasons. First, we have examined thousands of small mammal bones from the site^40^, and this bone tube fragment is unique with respect to polishing and surface modification. Had carnivores been common on the site, they would have surely left behind more than one piece of bone in their scats. Second, as discussed below, the artifact was manufactured from a skeletal element of low nutritional value that is often left unmodified by carnivores in the first place, suggesting that it would be a rare item even in an assemblage produced by carnivores^37,38^. Third, the context from which it was recovered, 1 m from a hearth feature in a dense scatter of cultural materials, strongly supports the hypothesis that humans made this artifact. It would require a remarkable series of events for the only carnivore-passed bone tube recovered from the site to be found in this location. Fourth, carnivore modification of faunal remains at La Prele is generally rare to non-existent, suggesting they were not present or at least kept away from the primary human living spaces at the site. Lastly, the grooves on the surface of the bead have U-shaped cross sections which can be produced by humans or carnivores^41,42^. While this does not eliminate the possibility that the grooves were created by gnawing, the grooves are fully consistent with creation by humans, either with stone tools or their own teeth. The collective evidence gives us confidence that the artifact is a human-modified bone bead.

## Results

**Figure 3:**
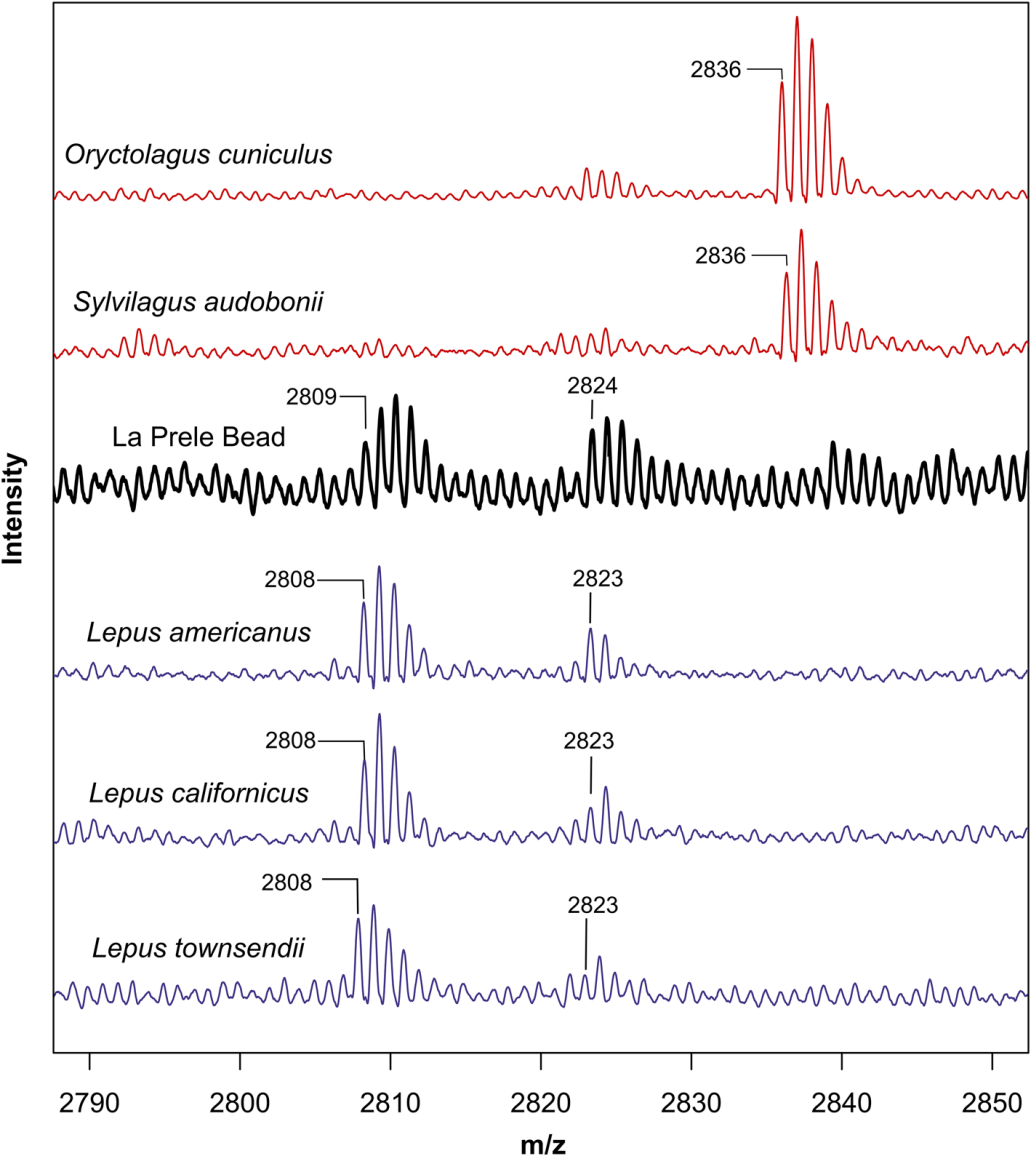
Partial MALDI-TOF MS spectra of the tryptic digests of bone collagen from the La Prele bead (thick black line) in comparison to modern rabbits (red) and hares (blue). The X-axis of the full spectra has been truncated to show m/z values from 2790 to 2850.

Using marker peptides from the bead MALDI-TOF spectra (Supplementary Data 1), we identified the material used to produce the bead as lagomorph bone, with greater similarity to hares (*Lepus*) than rabbits (*Oryctolagus*)^18,43^. However, given that the previously published markers were of European lagomorphs, we compared the bead spectra to those of common lagomorphs from the northwest Great Plains of North America using modern specimens in the zooarchaeology comparative collection at the University of Wyoming to gain greater confidence in taxonomic identification. The collagen proteins of North American taxa were not sequenced, so our identifications are based mostly on peptide mass comparisons. Nonetheless, peaks identified for North American rabbits and hares confirm previously identified differences in marker peptides for European lagomorphs^18,43^. Modern comparative specimens included three additional hares (black-tailed jackrabbit, white-tailed jackrabbit, and snowshoe hare) and two rabbits (desert cottontail and domestic rabbit) (Supplementary Data 2–6). The presence of a spectral peak at 2808.3 and the absence of a peak at 2836.3 positively identify the bead *Lepus* bone (Fig. 3; Supplementary Fig. 3). We are unable to achieve additional taxonomic specificity using these methods, but likely candidate species include the black-tailed jackrabbit (*L. californicus*), white-tailed jackrabbit (*L. townsendii*), snowshoe hare (*L. americanus*), or arctic hare (*L. arcticus*), which were present in Wyoming during the Pleistocene^44^. Hares have been recovered from several other early Paleoindian sites, although it is not clear if their presence is due to human behavior or natural factors^45–47^. Importantly, we also identified jackrabbit bone from another hearth-centered activity area (Block D) and rabbit bone from Block B^40^. To our knowledge, the bead reported herein is the first unambiguous evidence for the use of hares by Clovis foragers.

**Figure 4:**
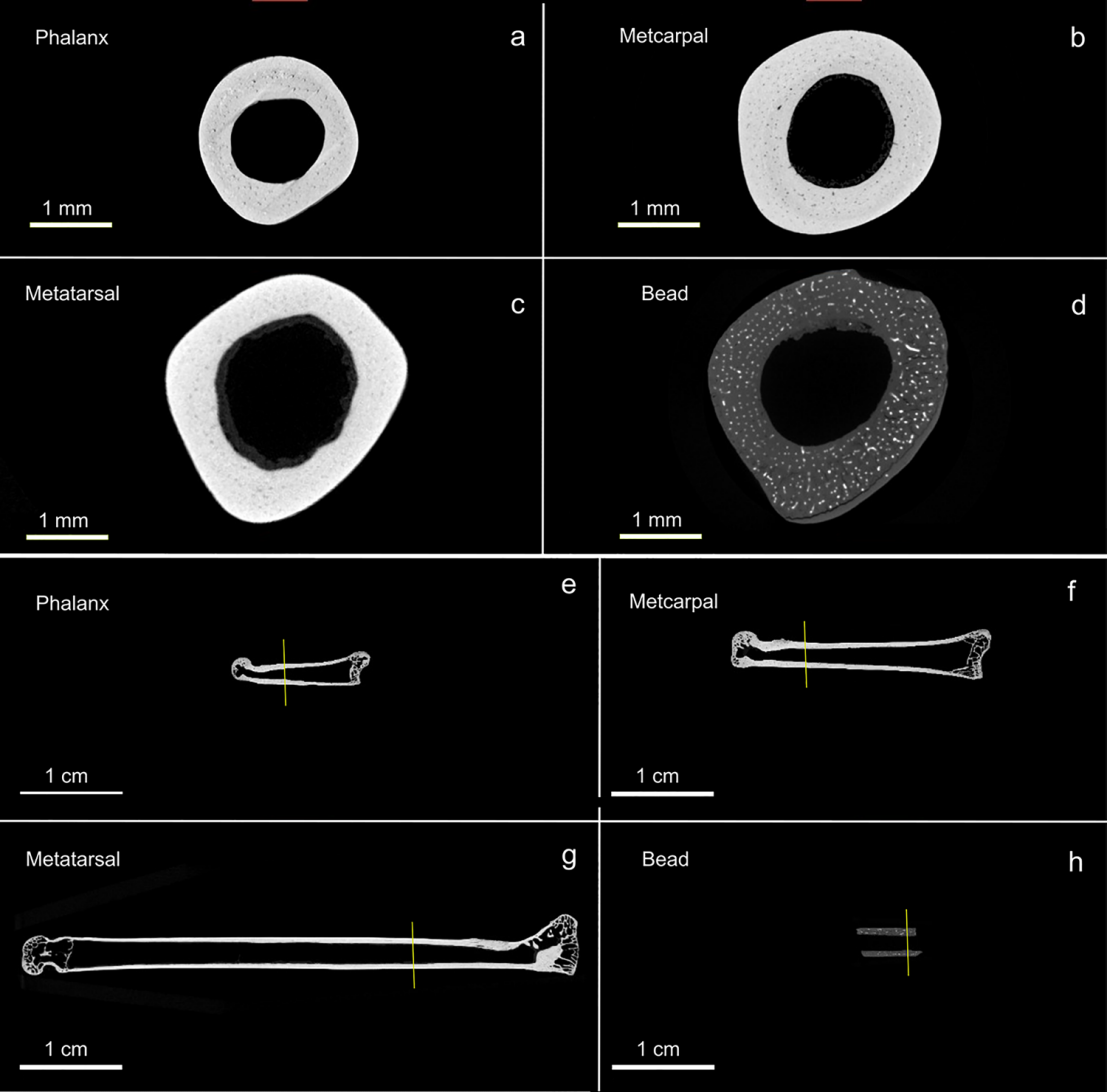
Micro-CT scans showing transverse (**a**–**d**) and longitudinal (**e**–**h**) cross sections of the La Prele bead (**d**,**h**) and a modern snowshoe hare proximal phalanx (**a**,**e**), metacarpal (**b**,**f**), and metatarasal (**c**,**g**). Yellow lines on longitudinal cross sections indicate the location of the transverse cross section for each specimen.

The lower limbs of rabbits and hares can furnish abundant raw material for producing tubular bone beads. For example, the metapodials and proximal and medial phalanges from a single animal can provide as many as 54 potential bead blanks^48^. Based on internal diameter, external diameter, thickness of the cortical bone, and the presence of a nontapering and parallel–sided medullary cavity, the bead was most likely manufactured from a metapodial (Fig. 4; Supplementary Fig. 4). Of the three comparative specimens we examined, the proximal portion of a metatarsal was the best overall match. Because the internal and external surface of the bead has likely been modified by wear, and allowing for significant intra- and interspecific morphological variation, we suspect that pinpointing the exact skeletal element used for its production will be extremely difficult.

## Discussion

There are many examples of the use of lagomorph bones for bead production in the Holocene archaeological records of Wyoming, Colorado, the Great Basin, and the Southwest^36,49–55^. We can now confidently say that this tradition extends back to the Early Paleoindian Period in the Rocky Mountain West. Importantly, tubular beads of hare bone are also known from sites in Paleolithic northeast Asia, including Denisova Cave and Yana RHS^35,56^. Groove and snap methods for bead production were used to manufacture *Lepus* bone beads in both Asia and North America^35,36^. While it is tempting to suggest that this practice came across the Bering Land Bridge with the first migrants to the Americas, more material evidence would be needed to fill the large spatiotemporal gap between Asian and North American examples. Independent innovation or diffusion seems equally plausible at this point.

There are long-standing debates about the extent to which Clovis foragers focused hunting efforts on large game or instead regularly incorporated a wide variety of plant and taxa^45–47,57–60^. Importantly, here we show an unambiguous example of the use of a small game taxon (*Lepus sp.*) during the Early Paleoindian period; the use of a metapodial or phalanx from a hare for the manufacture of a bead has little bearing on the question of what Clovis people hunted or ate. In fact, from the perspective of subsistence, this bone bead was found in close proximity to strong evidence for the subsistence use of *Bison antiquus* and *Mammuthus columbi*, the largest animals available in the region at the time. While small mammal remains are common in the stratum preserving the occupation at the site, there is little evidence that their presence is due to human activities^40^.

We hesitate to engage in extensive speculation as to the significance of bead use during Clovis times except to reiterate that beads and other personal ornaments and extrasomatic augmentations are most commonly used to signal aspects of identity to others^1^. Kuhn and Stiner^1^ hypothesize that the first appearance of beads in the Paleolithic might mark a time when population densities grew to the point where people were in regular contact with strangers. In the case of the bead from the La Prele Mammoth site, it is intriguing to note that during the Early Paleoindian period, when human population densities were low by any measure, beads were part of the Paleoindian cultural repertoire.

## Methods

To determine the type of animal used for the manufacture of the bead, we performed minimally destructive ZooMS analysis at the Ancient Biomolecules Laboratory at the University of Manchester and of modern taxa in the Geoarchaeology and Basile Laboratories at the University of Wyoming. Soluble collagen was extracted from the bead leaving it morphologically intact using a 0.3 M solution of HCl^61^, and then exchanged into 50 mM ammonium bicarbonate using a 10 kDa ultrafilter and digested with trypsin^62^. The archaeological spectra were produced using a Bruker Rapiflex MALDI TOF/TOF mass spectrometer. Modern comparative specimens for ZooMS were demineralized with 0.6 M HCl. Humic acids were removed with 0.1 M NaOH. Samples were gelatinized at 65 °C for one hour in ammonium bicarbonate and digested with trypsin as described above^18,20^. Analysis was performed with a Sciex 5800 MALDI-TOF mass spectrometer in the Basile Lab at the University of Wyoming. Archaeological spectra were compared to published^18^ and unpublished marker peptides for identification.

To identify possible skeletal elements used in the production of the La Prele bead, we used microcomputed tomography (micro-CT) scans to compare the morphology of a metacarpal, metatarsal, and proximal phalanx of a snowshoe hare to that of the La Prele bead. Scans were performed on a ZEISS Xradia 610 Versa using the following X-ray tube parameters: 60 kV, 108 µA and 6.5 W of power. Images were collected using a 0.4 × objective, a geometrical magnification of 8.5, a cone angle of 11.69, and a final pixel size of 8.05 µm. A total of 4501 projection images were acquired using 360 degrees of sample rotation. Scout and Scan™ Reconstructor (v16.1) was used to generate the final tomograms. All image analyses were performed using Dragonfly software (Comet Technologies, v2022.2).

### Supplementary Information

Supplementary Information 1.

Supplementary Information 2.

Supplementary Information 3.

Supplementary Information 4.

Supplementary Video 1.

Supplementary Information 5.

Supplementary Information 6.

Supplementary Information 7.

Supplementary Information 8.

Supplementary Information 9.

Supplementary Information 10.

Supplementary Information 10.

## Data Availability

All data generated or analysed during this study are included in this published article [and its supplementary information files].
