# Supplementary figures and images for: Use of hare bone for the manufacture of a Clovis bead
Source: Sci Rep. 2024 Feb 5;14:2937. doi: 10.1038/s41598-024-53390-9 (PMC10844228; doi:10.1038/s41598-024-53390-9)

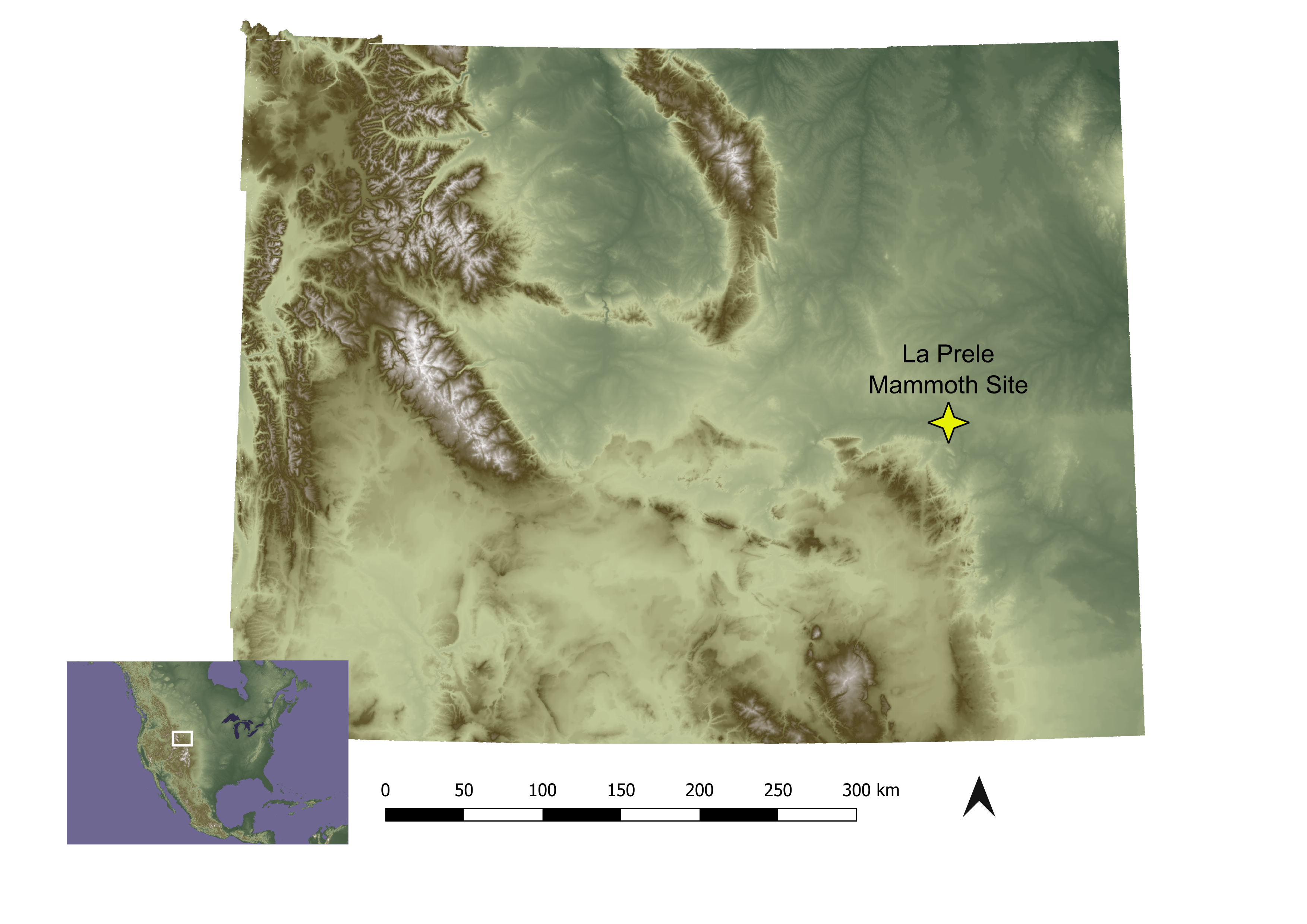

Supplement: Supplementary file 1 — Supplementary Information 1. [file 41598_2024_53390_MOESM1_ESM.png]

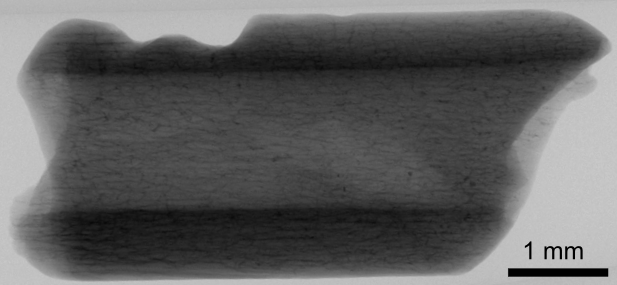

Supplement: Supplementary file 2 — Supplementary Information 2. [file 41598_2024_53390_MOESM2_ESM.png]

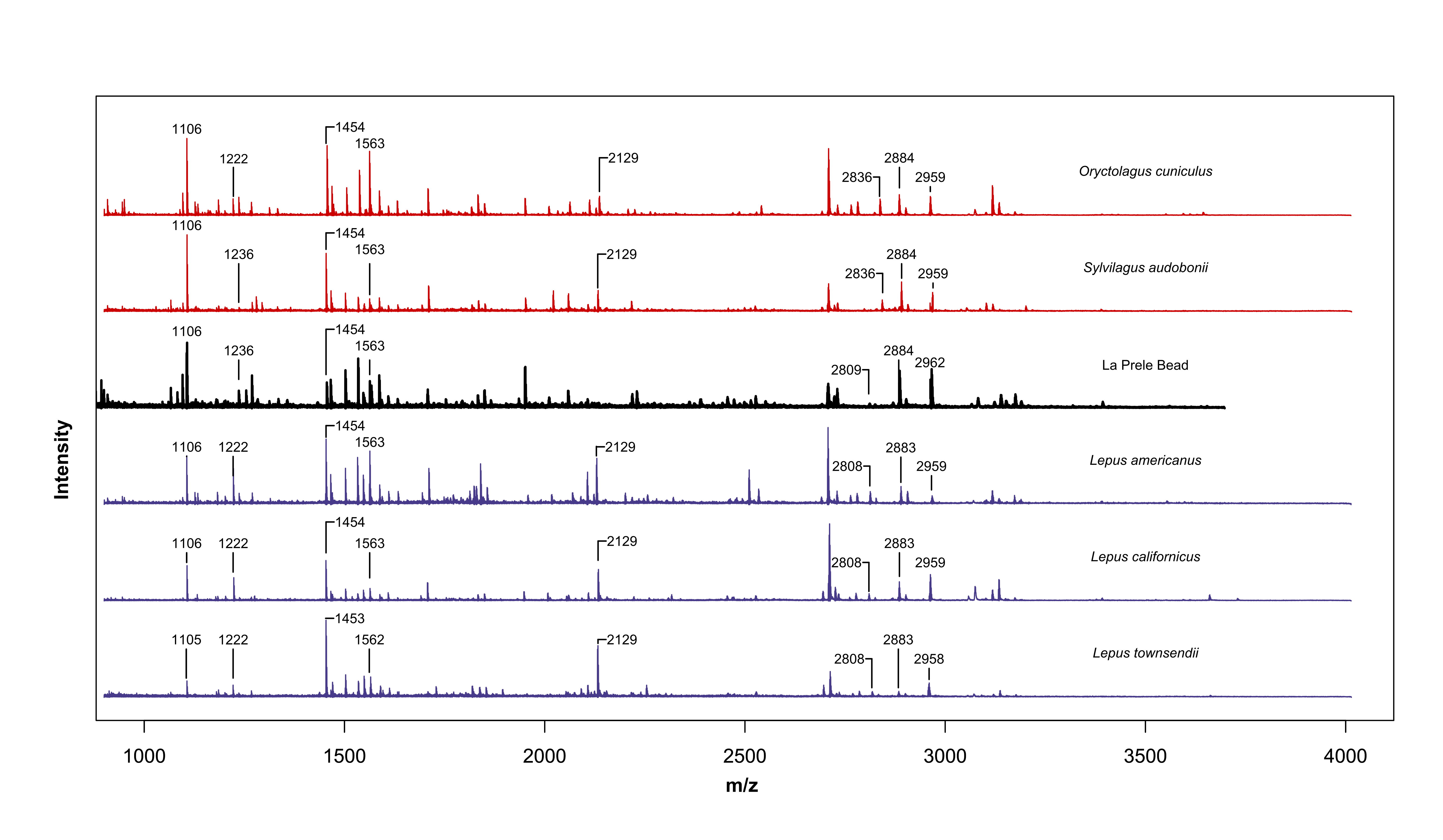

Supplement: Supplementary file 3 — Supplementary Information 3. [file 41598_2024_53390_MOESM3_ESM.png]

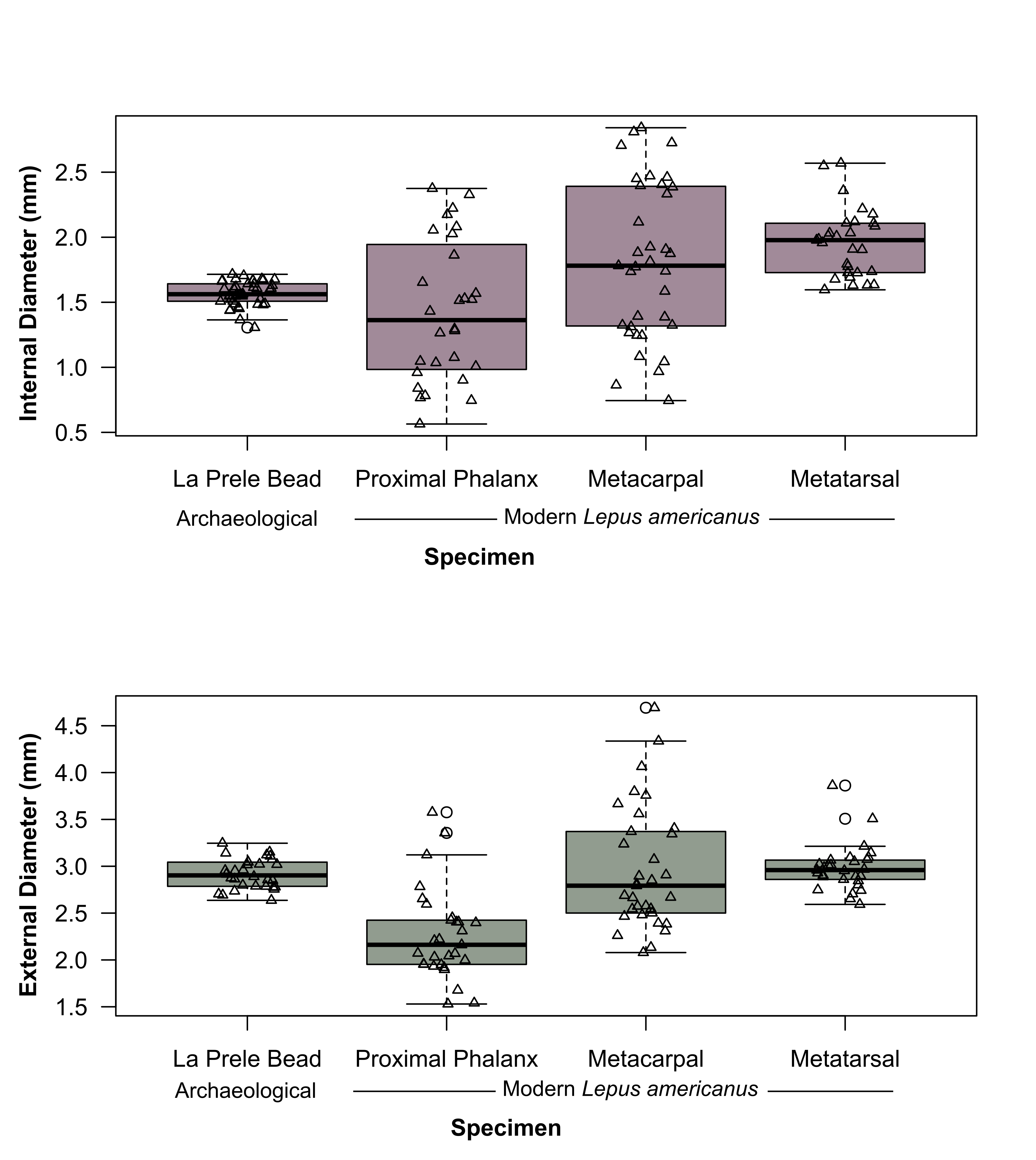

Supplement: Supplementary file 4 — Supplementary Information 4. [file 41598_2024_53390_MOESM4_ESM.png]
