# Supplementary material for: Use of hare bone for the manufacture of a Clovis bead
Source: Sci Rep. 2024 Feb 5;14:2937. doi: 10.1038/s41598-024-53390-9 (PMC10844228; doi:10.1038/s41598-024-53390-9)
Supplement: Supplementary file 12 — Supplementary Information 10. [file 41598_2024_53390_MOESM12_ESM.docx]

**Supplementary Figure and Video Captions**

Supplementary Figure 1. Location of the La Prele Mammoth site in Wyoming. Map created using QGIS, v 3.32^63^, and background map is a digital elevation model^64^.

Supplementary Figure 2. Micro-CT scan of the La Prele bead showing transverse grooves in cross section.

Supplementary Figure 3. MALDI-TOF MS spectra of the La Prele bead (black) and in comparison, to two species of rabbit (red) and three species of hare (blue). m/z values for marker peptides are labeled on each spectrum.

Supplementary Figure 4. Internal and external diameter measurements made from micro-CT scans of the La Prele bead in comparison to a metatarsal, metacarpal, and proximal phalanx of a modern snowshoe hare. Circles are outlying values; triangles show values of individual data points with randomized x-positions.

Supplementary Video 1. Animation of La Prele bead showing 3D morphology from micro-CT scans.

Supplementary Data 1. MALDI-TOF spectrum of the La Prele bead

Supplementary Data 2. MALDI-TOF spectrum of a modern snowshoe hare (*Lepus americanus*)

Supplementary Data 3. MALDI-TOF spectrum of a modern black-tailed jackrabbit (*Lepus californicus*)

Supplementary Data 4. MALDI-TOF spectrum of a modern white-tailed jackrabbit (*Lepus townsendii*)

Supplementary Data 5. MALDI-TOF spectrum of a modern domestic rabbit (*Oryctolagus cuniculus*)

Supplementary Data 6. MALDI-TOF spectrum of a modern desert cottontail rabbit (*Sylvilagus audobonii*)
